# *Necator americanus* and Helminth Co-Infections: Further Down-Modulation of Hookworm-Specific Type 1 Immune Responses

**DOI:** 10.1371/journal.pntd.0001280

**Published:** 2011-09-06

**Authors:** Stefan Michael Geiger, Neal Douglas Edward Alexander, Ricardo Toshio Fujiwara, Simon Brooker, Bonnie Cundill, David Joseph Diemert, Rodrigo Correa-Oliveira, Jeffrey Michael Bethony

**Affiliations:** 1 Centro de Pesquisas René Rachou, Fundação Oswaldo Cruz, Belo Horizonte, Minas Gerais, Brazil; 2 Department of Microbiology, Immunology and Tropical Medicine, Medical Center, The George Washington University, Washington, D.C., United States of America; 3 Department of Infectious and Tropical Diseases and Department for Epidemiology and Population Health, London School of Hygiene and Tropical Medicine, London, United Kingdom; 4 Departamento de Parasitologia, Instituto de Ciências Biológicas, Universidade Federal de Minas Gerais, Belo Horizonte, Minas Gerais, Brazil; Leiden University Medical Center, Netherlands

## Abstract

**Background:**

Helminth co-infection in humans is common in tropical regions of the world where transmission of soil-transmitted helminths such as *Ascaris lumbricoides*, *Trichuris trichiura*, and the hookworms *Necator americanus* and *Ancylostoma duodenale* as well as other helminths such as *Schistosoma mansoni* often occur simultaneously.

**Methodology:**

We investigated whether co-infection with another helminth(s) altered the human immune response to crude antigen extracts from either different stages of *N. americanus* infection (infective third stage or adult) or different crude antigen extract preparations (adult somatic and adult excretory/secretory). Using these antigens, we compared the cellular and humoral immune responses of individuals mono-infected with hookworm (*N. americanus*) and individuals co-infected with hookworm and other helminth infections, namely co-infection with either *A. lumbricoides*, *Schistosoma mansoni*, or both. Immunological variables were compared between hookworm infection group (mono- versus co-infected) by bootstrap, and principal component analysis (PCA) was used as a data reduction method.

**Conclusions:**

Contrary to several animal studies of helminth co-infection, we found that co-infected individuals had a further downmodulated Th1 cytokine response (e.g., reduced INF-γ), accompanied by a significant increase in the hookworm-specific humoral immune response (e.g. higher levels of IgE or IgG4 to crude antigen extracts) compared with mono- infected individuals. Neither of these changes was associated with a reduction of hookworm infection intensity in helminth co-infected individuals. From the standpoint of hookworm vaccine development, these results are relevant; i.e., the specific immune response to hookworm vaccine antigens might be altered by infection with another helminth.

## Author Summary

Parasitic infections in humans are common in tropical regions and under bad housing and sanitation conditions multiple parasitic infections are the rule rather than the exception. For helminth infections, which are thought to affect almost a quarter of the world's population, most common combinations include soil-transmitted helminths, such as hookworm, roundworm, and whipworm, as well as extra-intestinal infections by schistosomes. In order to develop and test a hookworm vaccine in endemic areas, the understanding of the impact of multiple helminth infections (co-infection) on the immune response against hookworm in infected individuals is crucial. The authors report in their article, that several parameters of the cellular (T cell markers, cytokines, chemokines) and humoral immune response (e.g. IgG4 and IgE antibodies) against hookworm are significantly affected or modulated in individuals co-infected with hookworm, roundworm and/or schistosomes. These results imply that the immune response against components of a hookworm vaccine might be altered by previous contact with other helminth species in endemic areas.

## Introduction

Helminth co-infection in humans is common in tropical regions [1], [2], where transmission of *Ascaris lumbricoides*, *Trichuris trichiura*, the hookworms (*N. americanus* or *A. duodenale*), and schistosomes often occur concurrently [3], [4]. Although co-infection is often the rule rather than the exception in endemic areas, most previous immuno-epidemiological studies of human helminth infection have focused on the immune response to a single helminth species (mono-infection) rather than the more common situation where an individual is infected with one or more different helminth species [5]. At our study site in Northeastern Minas Gerais State, Brazil, where co-infection with schistosomes and soil-transmitted helminths (STHs) is common [6], we have attempted to study the epidemiologic, immunologic, and genetic determinants of infection in individuals resident in these co-endemic areas [7]–[11].

Much of the previous information on the immunology of helminth co-infections has come from laboratory animal models, especially experimental rodent models. The majority of these studies show a competition between the co-infections, with one infection usually leading to the rapid expulsion of the other [12]–[16]. The immune mechanisms behind this effect are hypothesized to include cross-reactive antibodies (also referred to as “cross-protection”) [13], [16], a skewing towards Th2 cytokines (e.g., elevated IL-4), increased Th2-type antibody isotypes (e.g., elevated production of IgG1) [15], and mucosal mast cell activation [13]–[15]. However, conflicting animal studies report that co-infection increases infection intensities by down modulating Th2 cytokine responses, which in turn reduces intestinal inflammation, leading to slower worm expulsion and increased worm burdens in co-infected animals [17]. Possible explanations for these opposite findings, among others, might be differences in animal models, different combinations of parasite infections, and the different timing of co-infection (by timing of the primary versus the secondary infection).

The few studies on the human immune response in co-infected individuals are also contradictory. In one group of studies, helminth co-infection appeared to result in a synergistic effect among the infections, with infection with one helminth being associated with an increased risk of having a high intensity infection with another helminth [7]. However, other studies imply a cross-protective effect derived from co-infection: for example, individuals mono-infected with hookworm or *A. lumbricoides* develop antibodies that cross-react with antigens from *S. mansoni* [18]–[20]. In another set of studies, co-infection appeared to skew the immune response away from the helminth infection under study, e.g., the humoral and cellular immune responses to hookworm or *Ascaris* antigens are diminished in individuals resident in a schistosomiasis endemic area [21]. Along these same lines, studies have also demonstrated an upregulation of the immune response during helminth co-infection; e.g., increased production of inflammation markers to *S. mansoni* infection in children who are also infected with hookworms and/or *Entamoeba* species [22]. However, given the contradictory nature of these outcomes, the central question of whether multiple helminth infections drive host immune responses towards phenotypes different from those of a single infection still remains to be answered [23].

In our previous epidemiological study in Brazil, we showed synergistic effects among helminth co-infections in terms of egg counts [7], leading us to expect a similar synergistic effect on immune responses during helminth co-infection. In keeping with the results from experimental animal studies [12]–[16], we further hypothesized that hookworm co-infections with *A. lumbricoides* and/or *S. mansoni* would significantly alter the immune responses to crude hookworm antigen extracts, resulting in reduced Th2-type responses (IL-4, IL-5, IL-13), a reduced inflammatory response (e.g., lower TNF-α secretion), and an increase in the production of regulatory cytokines (e.g., IL-10). To test this hypothesis, we compared the cellular and humoral immune responses of individuals infected with hookworm alone (mono-infected) and individuals infected with hookworm and either *A. lumbricoides*, *S. mansoni* or both (co-infected).

## Materials and Methods

### Study site and selection of patients

The study was conducted in an area of the northeastern part of the state of Minas Gerais in Brazil that is endemic for *S. mansoni* and the STH as previously described [7]. The area of Americaninhas is divided into five rural sectors and a central municipality. The Fundação National de Saúde (the National Health Foundation) estimates the population to be approximately 1000 in the urban municipal center and another 1000 in the surrounding rural areas. Each house was assigned a unique household identification number (HHID), and each resident, a unique personal identity number (PID). Only individuals meeting the following inclusion criteria were included into the study: (1) resident in the study area over the last 24 months; (2) reporting not to have received anthelmintic treatment within the last 24 months; and (3) willing and able to give informed consent to study protocol. Individuals were not included if they: (1) attended school outside the study area; (2) worked full-time outside the study area; or (3) tested positive on a pregnancy test. Females found to be pregnant during the test were excluded from treatment during their pregnancy and received treatment for all helminth infections later. For parasitological exams, participants were instructed to deposit one fecal sample per day into each container and return the container to one of several collection points, where the sample was stored at 4°C. Fecal samples returned later than 48 h after date of distribution were not accepted, and new containers were issued. Presence of infection was determined by using the formalin-ether sedimentation technique. Individuals positive for any helminth in the formalin-ether sedimentation technique were asked to contribute two more samples over the course of two more days to be analyzed by Kato-Katz technique for assessment of eggs per gram of feces (infection intensity). Two slides were taken from each day's fecal sample for a total of four slides from each individual. Slides were examined within 45 minutes of slide preparation to avoid drying of hookworm eggs. The arithmetic means of the four slides was calculated and then converted to eggs per gram according to the Kato-Katz method [24].

Out of 1,332 consented participants in the study, two-hundred and fifty individuals were selected by simple random sampling for immunological assays. Random sampling was performed on an age, gender, and infection stratified sampling frame. In brief, individuals with a negative fecal exam were removed from the sampling frame; i.e., only persons with a positive fecal exam were included. The sampling frame was then divided into 10 mutually exclusive and exhaustive gender-based strata based using the following age intervals: <9, 10–19, 20–29, 30–39, and >40 years of age. Simple random sampling was performed independently in each stratum. Individuals who refused to enroll in this part of the study or who were not eligible were replaced by simple random sampling from the same stratum. The final stratified random sample was compared to non-participants for age, gender, and infection intensity, and no statistically significant differences (p>0.05) were found in terms of those variables between those individuals included in the survey and those not.

Individuals found to be infected with hookworm or other intestinal nematodes were treated with albendazole (400 mg). Participants with schistosomiasis were treated with praziquantel (50 mg/kg) under the supervision of the project physician.

In the present study, cellular and humoral immune responses from individuals with a hookworm mono-infection [9] were included, as well as from individuals co-infected with (a) hookworm and *A. lumbricoides*, (b) hookworm and *S. mansoni*, or (c) hookworm, *A. lumbricoides* and *S. mansoni*. After parasitological exams and before anthelminthic treatment, approximately 20 mL of blood was collected in heparinized tubes from children ≥6 years of age and adults for separation of peripheral blood mononuclear cells (PBMC) and 4 mL of blood in EDTA tubes for the immunological assays described below. The study was approved by the ethical review committees of The George Washington University (GWU, USA), the London School of Hygiene and Tropical Medicine (UK), the Centro de Pesquisas René Rachou FIOCRUZ and the Brazilian National Committee for Ethics in Research (CONEP), and all subjects provided written informed consent to participate in the study, or, in the case of minors, written informed consent was given by their parents or guardians.

### Phenotyping of lymphocytes *ex vivo*

Phenotyping of lymphocytes was performed as described elsewhere [9] and the following pairs of monoclonal antibodies (mAb), either conjugated with phycoerythrin (PE) or fluorescein isothiocyanate (FITC) were used: CD4(FITC)/CD25(PE), CD4(FITC)/HLA-DR(PE), CD4(FITC)/CD45RO(PE), CD4(FITC)/CD45RA(PE), CD8(FITC)/CD28(PE), CD8(FITC)/HLA-DR(PE), CD8(FITC)/CD45RO(PE), CD8(FITC)/CD45RA(PE), CD3(FITC)/CD69(PE), and CD19(FITC)/CD27(PE). Mouse IgG1 antibodies conjugated with FITC or PE served as isotype controls. Sample acquisition was done on a FACScan flow cytometer (Becton Dickinson, USA) and results for 10,000 events were analysed with BD Cell Quest™ software (Becton Dickinson, USA).

### Enzyme linked immunosorbant assays (ELISA) for antigen-specific antibody classes and sub-classes in serum samples

For the evaluation of humoral and cellular immune responses, soluble somatic antigen extracts were prepared from third-stage larvae (L3) and adult worms (AE) of *Ancylostoma caninum*. Excretory/secretory (ES) antigens were obtained from cultured *A. caninum* adult worms. The preparations were performed as described elsewhere [9]. For the detection of parasite-specific IgE antibodies, each of the hookworm antigens were diluted with carbonate buffer (pH 9.6) to a concentration of 5 µg/ml. High-binding ELISA plates (NUNC, Maxisorp, Fisher Scientific, USA) were coated with 100 µl of the diluted antigens and incubated overnight at 4°C. Plates were washed 5 times with washing buffer (phosphate buffered saline [PBS]/0.05% Tween-20; pH 7.2–7.4) and were then blocked for 1 hour at room temperature (RT) with 200 µl of blocking buffer (PBS/ 0.05% Tween-20/ 3% bovine serum albumin). Individual serum samples were diluted 1∶50 in blocking buffer, 200 µl were added in duplicate to the respective wells, and plates were incubated overnight at 4°C. On the following day, plates were washed 10 times with washing buffer. A 1∶1,000 dilution of anti-human IgE alkaline phosphatase-conjugated antibody (Pharmingen, USA) was prepared in PBS/0.05% Tween-20 and 100 µl were added to the wells. After another incubation of 90 minutes at RT, plates were washed 5 times and then 100 µl of p-nitrophenyl phosphate substrate was added to each well. Plates were incubated overnight at 4°C and the following morning the color reaction was read at 405 nm using an automated ELISA reader (SpectraMax 340 PC, Molecular Devices, USA) using SOFTmax Pro 5.2 for Windows (Molecular Devices) for data capture. Reference sera were assayed on each plate as positive and negative controls.

For detection of parasite-specific IgG subclasses, horseradish peroxidase-conjugated, anti-human IgG1, IgG3, and IgG4 (Zymed, USA) were used at a dilution of 1∶1000, as described above. As substrate, ortho-phenylene diamine was used and the color reaction was stopped with H_2_SO_4_ after incubation for 30 min at RT in the dark. Plates were read at 490 nm.

### Lymphocyte separation, proliferation assays and cytokine/chemokine secretion *in vitro*

The separation of lymphocytes, their stimulation *in vitro* with different hookworm antigens and with the mitogen phytohemagglutinin (PHA), lymphocyte proliferation, as well as the secretion of several cytokines and chemokines after *in vitro* stimulation were performed as described elsewhere in detail [9]. Here we report the proliferation of lymphocytes after stimulation with the crude soluble hookworm antigens L3, AE, and ES. For *in vitro* cytokine or chemokine secretion, lymphocyte cultures were stimulated with the same antigens and with PHA, as described for proliferation assays, and the following analytes were measured: Interleukin (IL)-2, IL-4, IL-5, IL-10, IL-13, CXCL10, TNF-α, and IFN-γ.

### Statistical analyses

**Figure 1:**
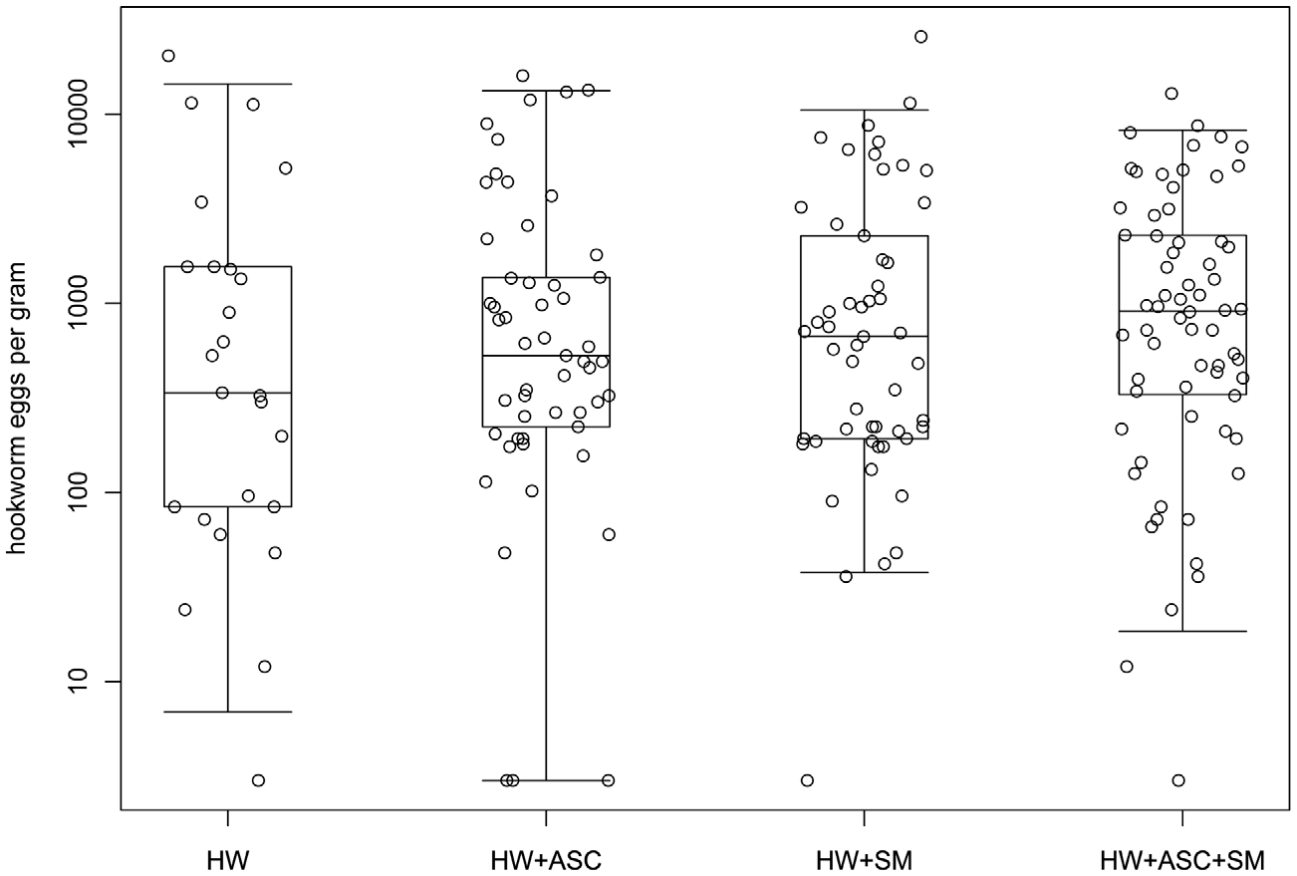
Fecal egg counts in hookworm mono- and co-infected individuals. *Footnotes:* Circles represent individual values for eggs per gram of feces (epg) and are shown on a logarithmic scale. Boxes indicate the median and the quartiles for each group and the whiskers indicate the 95% ranges. Groups are split in hookworm mono-infected (HW), co-infected with *A. lumbricoides* (HW+ASC), co-infected with *S. mansoni* (HW+SM), and triple-infected individuals (HW+ASC+SM). Kruskal-Wallis test on differences in hookworm egg counts between groups was not statistically significant (*p* = 0.523).

The intensity of hookworm infection (as determined by fecal egg counts) was compared between groups by non-parametric Kruskal-Wallis test. Associations between *Necator* intensity of infection and antibody level against crude antigen extracts or *Necator* infection intensity and secreted cytokines/chemokines were analysed by Spearman's rank correlation. Analyses of these immune responses were done separately for the different co-infection combinations and then compared with hookworm mono-infected individuals. As the results among the different co-infection subgroups were found to be generally similar (see below, in particular Table 1 and Figure 1), we merged the various co-infections into a single group. For the chemokine and cytokine variables, analysis was done on the log-transformed variables, after replacing any zero values with 1. Immunological variables were compared by bootstrapping the geometric mean after adjusting for age by linear regression on the log-values. For the lymphocyte populations, the untransformed values were used and hence the arithmetic means were compared. The immunological variables were summarized using principal component analysis (PCA), via a projection-pursuit algorithm robust to departures of the data from normality [25], [26]. We then used biplots [27] to simultaneously show i) the contributions of each of the original variables to the first two principal components (the ‘loadings’), and ii) each person's value of the principal components (the ‘scores’). The bivariate score means and their 95% confidence ellipses [28] were calculated for the mono-infected and co-infected groups. These means were compared between infection groups by the multivariate Hotelling's *T* ^2^ test [29]. PCA analysis was done for lymphocyte sub-populations, for antibody responses, and for chemokine and cytokine response to three hookworm antigen preparations (AE, ES, and L3) and a mitogen (PHA) Pairs of correlation coefficients by infection group were compared by first transforming the variable to a standard normal deviate via the Fisher *Z* transformation. No adjustment for multiple comparisons was made in these analsyses. Analyses were performed using S-PLUS version 6.2 or later (Insightful Corp, Seattle WA, USA) and R version 2.10 or later (R Foundation for Statistical Computing, Vienna, Austria). The PCA analysis used the ‘pcaPP’ package in R.

**Table 1:** Demographic characteristics of groups mono- or co-infected with hookworm.

Patient groups	HW	HW+ASC	HW+SM	HW+ASC+SM
Number of individuals	25	53	53	66
Males/females	16 / 9	21 / 32	33 / 20	33 / 33
Median age (range)	53	36	42	31
	(15–70)	(6–76)	(8–74)	(7–83)
Median HW epg	366	528	666	909
(range)	(3–20,376)	(3–15,978)	(3–25,698)	(3–12,864)
Median ASC epg	0	6,012	0	2,403
(range)	0	(3–12,024)	0	(3–12,024)
Median SM epg	0	0	72	99
(range)	0	0	(3–1,122)	(3–3,774)

*Footnotes:* Indicated are the total number of participants, numbers of males and females, median age and median egg counts per gram feces (epg) in individuals mono- and co-infected with hookworm. Abbreviations: HW: hookworm; ASC: *A. lumbricoides*; SM: *S. mansoni*. Hookworm mono-infected **(HW)**; patients co-infected with *A. lumbricoides* **(HW+ASC)**; co-infected with *S. mansoni* **(HW+SM)**; triple-infected patients **(HW+ASC+SM)**.

## Results

Of the 250 study participants who were randomly selected, 197 were infected with hookworm and were therefore included in the immunological assessments. Table 1 shows the demographic characteristics of individuals either mono-infected with *N. americanus*, co-infected either with *A. lumbricoides* or *S. mansoni*, or infected with all three helminth species. The median age in the co-infected groups was lower than in the mono-infected group, but the hookworm parasite load, estimated by the number of eggs per gram of feces, did not differ significantly between the four groups (Table 1 and Figure 1). Figure 1 shows the median fecal egg counts for the different groups, which covered a wide range of infection intensity.

### Phenotyping of lymphocytes

We observed a statistically significant increase in CD4/HLA-DR and CD8/HLA-DR positive T-cells in co-infected individuals compared to mono-infected individuals. Other comparisons of surface markers on T and B cells between mono- and co-infected individuals were not significant (see Table 2). PCA was performed on these immunological parameters jointly in order to obtain a more complete and integrated picture of the immunological pattern and compare the weight of each parameter's contribution to the immune response. The first principal component (PC 1) was dominated by a contrast between CD4^+^/CD25^+^ (positive loading) and CD8^+^/CD28^−^ T cells (negative loading). PC 2 is effectively an average of CD4/CD45RA and CD8/CD45RA positive memory T cells (see Figure S1).

**Table 2:** Cell surface markers on PBMC from hookworm mono- and co-infected individuals.

			Difference (adjusted for age)	95% confidence interval	*p*-value#
Population (n missing)	Hookworm mono-infected, n = 25,$	Co-infected, n = 189,$			
CD19/CD27 (1)	2.8	3.5	−0.6	(−1.8–0.2)	0.17
CD3/CD69 (0)	2.6	3.4	−0.7	(−1.8–0.5)	0.22
CD4/CD25 (11)	10.7	9.6	0.6	(−2.1–3.8)	0.66
CD4/CD45RA (16)	14.4	13.8	2.0	(−0.8–5.3)	0.16
CD4/CD45RO (0)	21.6	18.7	1.7	(−1.3–4.8)	0.26
CD4/HLA-DR (0)	1.4	2.3	−1.0	(−1.5–−0.5)	**<0.001**
CD8/CD28 (1)	9.4	10.4	−0.4	(−1.9–0.9)	0.60
CD8/CD28^neg^ (4)	21.0	16.9	2.6	(−1.9–7.4)	0.28
CD8/CD45RA (1)	19.8	18.0	1.7	(−1.8–5.4)	0.35
CD8/CD45RO (0)	6.2	5.6	0.1	(−1.5–1.8)	0.92
CD8/HLA-DR (22)	1.5	2.4	−1.1	(−1.8–−0.3)	**0.01**

*Footnotes:*

# Statistically significant differences between groups are highlighted in bold numbers.

$ Values indicate the arithmetic mean of the percentage of positive cells.

### Antigen-specific antibodies in mono- and co-infected patients

In participants either mono-infected or co-infected, we found positive correlations between individual fecal egg counts and serum IgG4 antibody levels against all the hookworm crude antigen preparations tested: L3, AE and ES. Other isotypes, such as IgG1, IgG3, and IgE, were not strongly correlated with egg counts (see Table S1). For individuals with co-infections, the correlations between fecal hookworm egg counts and hookworm-specific IgG4 were significant for AE (rho = 0.40; *p*<0.001), ES (rho = 0.21; *p* = 0.007), and L3 (rho = 0.26; *p* = 0.001) antigen preparations.

Optical density values for hookworm-specific serum antibodies were measured and the age-adjusted ratio between mono- and co-infected individuals are shown in Table 3, where we observed significantly higher values for L3-specific IgG3, IgG4, and IgE, AE-specific IgG1, IgG4, and IgE, and ES antigen specific IgG1 and IgG4 responses in co-infected individuals compared to mono-infected individuals (Table 3).

**Table 3:** Comparison of hookworm-specific antibody responses in sera from mono- and co-infected individuals.

				Ratio (adjusted for age)	95% confidence interval	*p*-value#
Antigen	Antibody classes and sub-classes (n missing)	Hookworm mono-infected, n = 25,$	Co-infected, n = 195,$			
L3	IgG1 (17)	0.40	0.54	0.79	(0.62–1.01)	0.06
	IgG3 (17)	0.13	0.14	0.91	(0.85–0.98)	**0.02**
	IgG4 (17)	0.18	0.24	0.76	(0.63–0.98)	**0.04**
	IgE (7)	0.19	0.29	0.65	(0.54–0.78)	**<0.001**
AE	IgG1 (16)	0.14	0.20	0.75	(0.61–0.93)	**0.01**
	IgG3 (16)	0.18	0.24	0.74	(0.54–1.05)	0.09
	IgG4 (16)	0.12	0.19	0.61	(0.52–0.74)	**0.002**
	IgE (7)	0.25	0.51	0.51	(0.39–0.68)	**<0.001**
ES	IgG1 (17)	0.13	0.16	0.82	(0.73–0.93)	**0.002**
	IgG3 (17)	0.14	0.16	0.86	(0.69–1.13)	0.25
	IgG4 (17)	0.10	0.11	0.87	(0.79–0.97)	**0.02**
	IgE (7)	0.24	0.29	0.86	(0.67–1.16)	0.30

*Footnotes:* Indicated are geometric mean optical density values, the age-adjusted ratio between mono-infected and co-infected individuals, the 95% confidence intervals, and the calculated *p*-values for statistical differences.

# Statistically significant differences between groups are highlighted in bold numbers.

$ Values indicate geometric mean values of optical densities.

**Figure 2:**
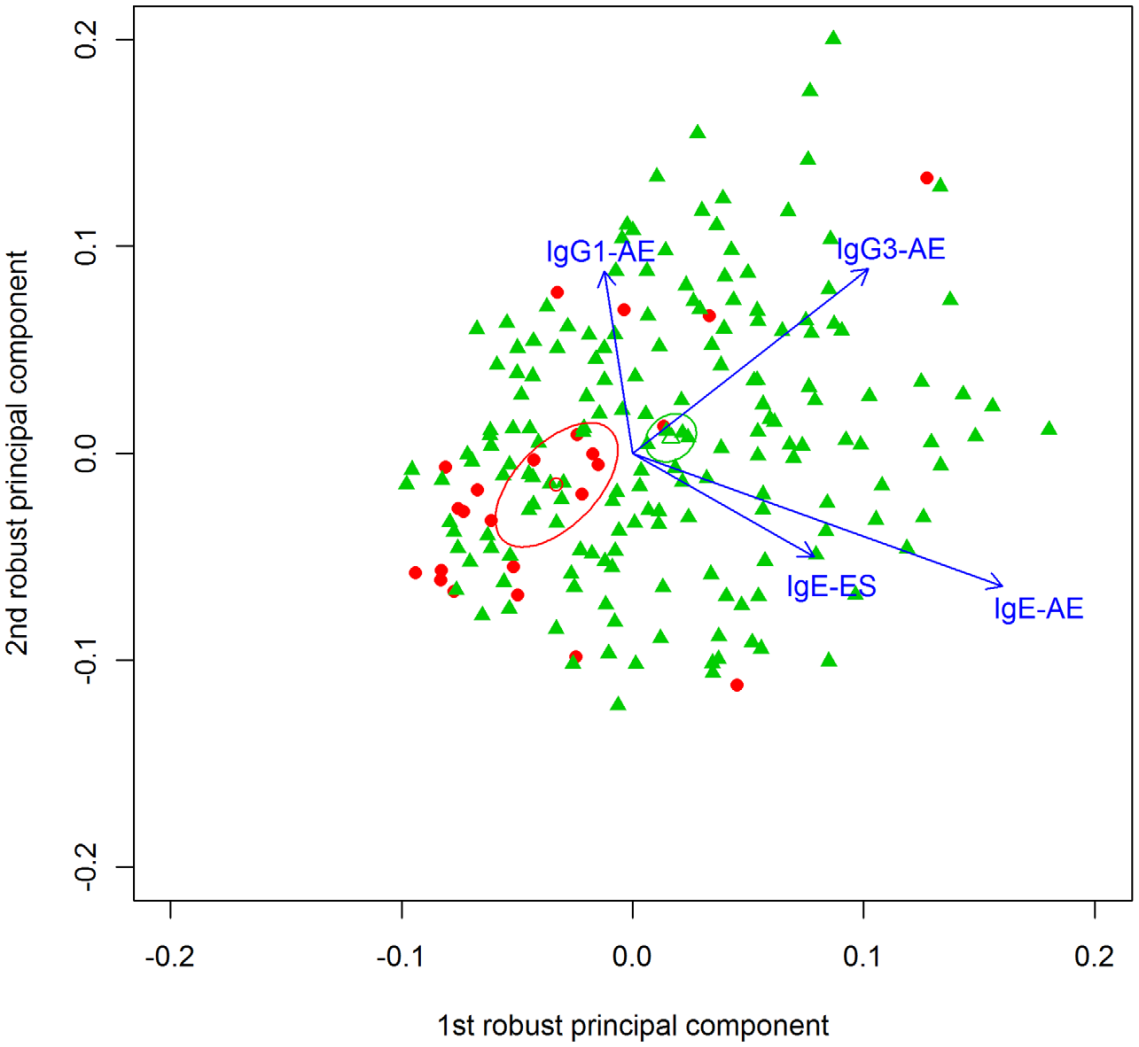
Robust principal component analysis (PCA) of log-transformed serum antibody values in response to hookworm antigens. *Footnotes:* The principal component scores for individuals mono- (•) and co-infected (▴) with hookworm are shown. The respective mean values are shown as open symbols, with 95% confidence ellipses (*p* value for bivariate *T*2 test is 0.006). The arrows show the strongest loadings, i.e. contributions of the original variables to the principal components.

Mean PC values for mono-infected and co-infected individuals, plus their 95% confidence intervals (ellipses), showed distinct segregation between these infection groups, with the mono-infected individuals having lower values of PC 1, which was dominated by IgG3 against AE antigen, and IgE against AE and ES antigens (Figure 2). PC 2 showed a contrast between i) IgG1 and IgG3 against AE antigen (positive loadings) and ii) IgE against AE and ES antigens (negative loadings).

### Lymphocyte proliferation and chemokine and cytokine responses

Values for lymphocyte proliferation were indicated as stimulation indices, i.e. proliferation of antigen- or mitogen-stimulated cells divided by the proliferation of unstimulated control cultures. Analysis of lymphocyte proliferation did not result in any significant differences between mono- and co-infected groups (data not shown). Non-parametric correlations between individual PBMC secreted cytokine or chemokine levels and fecal hookworm egg counts were strongly negative for IL-10 in mono-infected participants and significantly different when compared with co-infected individuals, whether stimulated with L3 or AE (*p* = 0.032 for both comparisons), or ES antigen (*p* = 0.003, Table 4). Likewise, strong negative correlations were found for TNF-α in control cultures from mono-infected individuals or when cells were stimulated with ES, which were significantly different from the co-infected group (*p* = 0.002 and *p* = 0.04, respectively, Table 4). In individuals with co-infection, significant negative correlations between egg counts and CXCL10 secretion were found in cell cultures stimulated with L3 (*p*<0.05) or ES antigen (*p*<0.01), however without any significant differences when compared with mono-infected individuals.

**Table 4:** Correlations (Spearman's rank test) between individual hookworm egg counts and antigen-induced cytokine/ chemokine secretions.

Cytokine/chemokine	Hookworm antigen	Mono-infected	Co-infected	*p*-value for differences between groups
IL-10	Control	−0.43	−0.06	0.142
	(*p*-value)	(0.075)	(0.512)	
	L3	**−0.55** *	−0.04	**0.032** *
	(*p*-value)	**(0.018)**	(0.690)	
	AE	**−0.49** *	0.06	**0.032** *
	(*p*-value)	**(0.042)**	(0.523)	
	ES (*p*-value)	**−0.73** **	−0.05	**0.003** **
	(*p*-value)	**(0.001)**	(0.594)	
CXCL10	Control	−0.30	0.00	0.222
	(*p*-value)	(0.200)	(0.964)	
	L3	−0.10	**−0.21** *	0.681
	(*p*-value)	(0.662)	**(0.011)**	
	AE	0.21	−0.03	0.337
	(*p*-value)	(0.371)	(0.703)	
	ES	0.00	**−0.26** **	0.350
	(*p*-value)	(0.994)	**(0.003)**	
TNF-α	Control	**−0.66** **	−0.09	**0.006** **
	(*p*-value)	**(0.002)**	(0.288)	
	L3	−0.25	−0.14	0.667
	(*p*-value)	(0.298)	(0.090)	
	AE	−0.23	−0.04	0.446
	(*p*-value)	(0.338)	(0.671)	
	ES	**−0.52** *	0.00	**0.040** *
	(*p*-value)	**(0.033)**	(0.960)	

*Footnotes:*

*correlation significant at the 0.05 level (2-tailed).

**correlation significant at the 0.01 level (2-tailed).

Analysis of cytokine and chemokine production in PBMC after stimulation with L3 antigen resulted in a significantly higher production of CXCL10 in mono-infected individuals (Table 5). Also, in PBMC stimulated either with AE or ES crude antigen extracts, significantly higher concentrations of TNF-α or IFN-γ were observed in mono-infected individuals when compared with the co-infected group (Tables 6 and 7). Examples of PCA for antigen-specific cytokine and chemokine secretion are shown in Figures S2, S3. For AE, as well as for ES antigen stimulation of PBMC, the highest loadings for PC1 and PC2 with the same directions were obtained for both Th1- and Th2-type cytokines or chemokines.

**Table 5:** L3 antigen-induced cytokine and chemokine secretion in lymphocyte cultures from individuals mono- or co-infected with hookworm.

	Mean value (pg/ml)	Mean value, (pg/ml)	Ratio (adjusted for age)	95% confidence interval	*p*-value#
Cyto- or chemo-kine (n missing, n below detection threshold)	Hookworm mono-infected, n = 23,$	Co-infected, n = 186,$			
IL-2 (16, 46)	9.0	10.7	0.85	(0.43–1.62)	0.65
IL-4 (16, 44)	6.9	8.3	0.91	(0.46–1.71)	0.77
IL-5 (46, 22)	31.1	41.2	0.85	(0.30–2.17)	0.74
IL-10 (46, 3)	363	240	1.56	(0.91–2.59)	0.11
IL-13 (1, 19)	136	98	1.47	(0.79–2.32)	0.21
CXCL10 (16, 18)	110	48	2.29	(1.24–4.30)	**0.01**
TNF-α (16, 10)	56.6	37.7	1.61	(0.88–2.91)	0.12
IFN-γ (47, 19)	214	139	1.56	(0.44–4.7)	0.46

*Footnotes:*

$ Indicated are geometric mean concentrations (pg/ml) for both groups, together with the age-adjusted ratios between groups and the 95% confidence intervals for L3 antigen preparation.

# Statistically significant differences between groups are highlighted in bold numbers.

**Table 6:** Adult worm antigen-induced cytokine and chemokine secretion in lymphocyte cultures from individuals mono- or co-infected with hookworm.

	Mean value, (pg/ml)	Mean value, (pg/ml)	Ratio (adjusted for age)	95% confidence interval	*p*-value#
Cyto- or chemo-kine (n missing, n below detection threshold)	Hookworm mono-infected, n = 22,$	Co-infected, n = 186,$			
IL-2 (15, 42)	12.5	12.7	1.03	(0.51–1.87)	0.94
IL-4 (15, 43)	7.1	8.0	0.92	(0.47–1.70)	0.78
IL-5 (47, 34)	29.9	25.8	1.33	(0.50–3.22)	0.56
IL-10 (47, 15)	65.4	60.4	1.24	(0.39–3.39)	0.70
IL-13 (0, 22)	65.3	63.7	1.12	(0.41–2.60)	0.80
CXCL10 (15, 52)	27.7	17.3	1.61	(0.58–4.31)	0.35
TNF-α (15, 19)	46.4	21.0	2.20	(1.38–3.74)	**<0.001**
IFN-γ (47, 44)	146.3	32.3	4.88	(1.33–15.9)	**0.02**

*Footnotes:*

$ Indicated are geometric mean concentrations (pg/ml) for both groups, together with the age-adjusted ratios between groups and the 95% confidence intervals for adult antigen preparation (AE).

# Statistically significant differences between groups are highlighted in bold numbers.

**Table 7:** ES antigen-induced cytokine and chemokine secretion in lymphocyte cultures from individuals mono- or co-infected with hookworm.

	Mean value, (pg/ml)	Mean value, (pg/ml)	Ratio (adjusted for age)	95% confidence interval	*p*-value#
Cyto- or chemo-kine (n missing, n below detection threshold)	Hookworm mono-infected, n = 16,$	Co-infected, n = 167,$			
IL-2 (15, 43)	8.6	9.8	0.85	(0.37–1.80)	0.68
IL-4 (15, 40)	5.7	7.3	0.79	(0.38–1.66)	0.55
IL-5 (42, 50)	4.6	8.2	0.56	(0.21–1.88)	0.33
IL-10 (42, 13)	175	80	2.34	(0.72–5.97)	0.14
IL-13 (0, 37)	26.7	28.7	1.01	(0.29–3.20)	0.98
CXCL10 (15, 78)	3.1	4.5	0.68	(0.28–1.99)	0.44
TNF-α (15, 8)	73.6	45.3	1.66	(1.05–2.76)	**0.03**
IFN-γ (42, 39)	69.6	26.7	2.64	(1.28–5.37)	**0.01**

*Footnotes:*

$ Indicated are geometric mean concentrations (pg/ml) for both groups, together with the age-adjusted ratios between groups and the 95% confidence intervals for ES antigen preparation.

# Statistically significant differences between groups are highlighted in bold numbers.

## Discussion

This is the first study to comprehensively examine the hookworm-specific humoral and cellular immune response in individuals who are co-infected with other helminths in an area of high hookworm transmission. This is also the first study to examine the effect of co-infection on the immune response to crude hookworm antigen extracts from different stages of hookworm development (L3, AE, ES). Moreover, these effects were analyzed in an epidemiologically well-characterized group of individuals, where the spatial, genetic and demographic aspects of hookworm infection and co-infection have been intensively studied [7], [8], [10], [11]. Apart from non-parametric methods and comparisons of individual parameters, we also utilized principal component analysis for comparison of the immune responses to hookworm crude antigen extracts between mono- and co-infected individuals, enabling us to examine, and compare numerous mutually correlated immune variables in relation to the effects of mono- or co-infection status [30].

Our analyses showed that chronic co-infection with nematode and trematode species considerably alters the immune response to hookworm crude antigen extracts. Most interestingly, co-infection altered to a significant degree the antigen-induced secretion of inflammatory TNF-α and led to a further diminution of hookworm-specific IFN-γ and CXCL10 secretion, but did not alter production of IL-10 or the Type-2 cytokines, when compared to mono-infected individuals. In contrast to our previous study [9], we found that the immune response to hookworm infection was increasingly modulated in co-infected individuals, an alteration that did not lead to expulsion of one parasite species as shown in experimental co-infections of mice with *S. mansoni* and *Trichuris muris* [15].

These findings are extremely relevant for successful planning of a hookworm vaccine currently under development [31]. In areas endemic for hookworm, such as the one studied, co-infections with other helminth species like *A. lumbricoides* and *Schistosoma* are common. Our results show that Type 1 immune responses to hookworm are significantly altered by such co-infections, which might have implications for hookworm vaccine development, with recent hookworm vaccines focused on inducing a Th1 response [32] in order avoid problems with hookworm induced IgE.

The major emphasis of our immunological study was on T cells, i.e., the proliferation of T cells, activation of T cell subpopulations, and secretion of Th1- and Th2-type cytokines and chemokines. Changes in CD4 and CD8 T cell counts, together with increased activation of these T cell subpopulations, have already been reported for helminth infections [33]. We add to this literature the finding that percentages of activated CD4^+^ and CD8^+^ T cells increased with co-infection. We speculate that multiply-infected individuals have higher percentages of activated CD4^+^ and CD8^+^ T cells due to ongoing higher antigenic stimulation of the immune system by different helminth species and cross-reactive antigens. This is supported by *in vitro* experiments on naïve human PBMC stimulated with soluble egg antigen from *S. mansoni* (SEA), which showed an increase in the CD4^+^/HLA-DR^+^ cell population after *in vitro* priming and a further increase during recall responses [34].

Even though mean fecal egg counts in mono-infected patients were found to be in the range of those from co-infected individuals, correlations between hookworm egg counts and hookworm-specific IgG4 responses were stronger in co-infected patients, which might be attributed to the presence of antibodies that were cross-reactive with antigens from co-infecting helminth species [21], [35], [36]. Chronic infections with multiple helminth species might induce a stronger and ongoing antigenic stimulation of the host's immune system, which may lead to the expansion of antigen-specific B cells and the secretion of specific IgG4 antibodies, especially in co-infected individuals with increased hookworm infection. In support of this, a prior study with volunteers co-infected with hookworm, *S. mansoni*, and *A. lumbricoides* showed an increase in helminth antigen-specific total IgG antibodies when compared with the respective mono-infected groups [21]. In hookworm infections, the production of all antigen-specific IgG subclasses rises with ongoing infection [35] and hookworm-specific IgG4 has been proposed as a good marker for patent and chronic infections [35]–[37].

Analysis of cytokine and chemokine secretion patterns from mono-infected volunteers revealed no clear polarization into Th1 or Th2 type immune responses, but rather a mixed pattern [9]. Similar results were recently obtained for individuals co-infected with *A. lumbricoides* and *T. trichiura* [38]. However, in the co-infected group, we found a decreased TNF-α secretion, together with a further down-modulation of hookworm-specific IFN-γ production. Another study on co-infection detected elevated levels of pro-inflammatory cytokines and chemokines in co-infected children in response to *S. mansoni* adult worm antigen, whereas IFN-γ and IL-13 secretion patterns revealed no significant differences between individuals mono- and poly-infected with schistosomes, hookworm and *Entamoeba* species [22]. As opposed to *A. lumbricoides* and *Trichuris trichiura* co-infections [38], we were neither able to detect a positive relationship between hookworm antigen-induced IL-10 secretion and intestinal worminess, nor to detect negative associations between IL-10 and Th1/Th2-type cytokines. These described differences might be due to the presence of different parasite species and also due to a mixture of intestinal and extra-intestinal parasites.

Considerable antigen-induced IL-10 secretion has been described in individuals with hookworm infection [9], [39]. In the current study, IL-10 levels correlated inversely with fecal egg counts in mono-infected hookworm patients especially in response to ES. This strong negative correlation was ablated in co-infected individuals, most probably because *A. lumbricoides* and *S. mansoni* infections induce production of IL-10 themselves [39]. Even though there was an unexpected negative correlation between parasite load and IL-10 secretion of lymphocytes, the antigen-induced IL-10 secretion was significantly associated with mono-infected individuals, indicating its importance in immune regulation during hookworm infection.

This study has some important limitations. First, the cross-sectional study design, in which groups are compared from a single time point, does not allow causal inferences to be made. In addition, the small sample size may have limited our ability to detect small statistical differences between groups. Nor does the sample size allow for further stratification of the groups in order to explore other factors which may account for these differences. Age is likely to be among the most important of such confounding factors but was included as a covariate when testing for differences between groups. One positive aspect of the study design was the population-based sampling which should enhance the generalizability of the study.

In summary, individuals co-infected with other helminth species presented with a significantly different immune response when compared with mono-infected participants. These changes included a stronger activation of CD4^+^ and CD8^+^ T cells, lower secretion of Type 1 cytokines, and increased levels of IgG4 and IgE antibodies against somatic hookworm antigens (L3 and AE). Furthermore, positive correlations between egg counts and hookworm-specific IgG4 responses, as well as missing correlations between egg counts and regulatory (IL-10) and inflammatory (TNF-α) cytokines in co-infected individuals. This modulation of hookworm-specific cellular and humoral immune responses by co-infection with other helminth species will be an important consideration during clinical trials for hookworm vaccine testing. Although vaccination is obviously not the same as natural infection, the immunogenicity of hookworm antigens in a vaccine might be altered and adversely affected by infections with parasites such as *S. mansoni* and *A. lumbricoides*.

## Supporting Information

Figure S1 **Robust principal component analysis (PCA) of** ***ex vivo*** **lymphocyte cell surface markers in PBMCs.** *Footnotes:* The principal component scores for individuals mono- (•) and co-infected (▴) with hookworm are shown. The respective mean values are shown as open symbols, with 95% confidence ellipses (*p* value for bivariate *T*2 test is 0.23). The arrows show the strongest loadings, i.e. contributions of the original variables to the principal components.
(TIF)

Figure S2 **Robust principal component analysis (PCA) of log-transformed cytokine and chemokine secretion in PBMCs stimulated with AE antigen.** *Footnotes:* The principal component scores for individuals mono- (•) and co-infected (▴) with hookworm are shown. The respective mean values are shown as open symbols, with 95% confidence ellipses (*p* value for bivariate *T*2 test is 0.13). The arrows show the strongest loadings, i.e. contributions of the original variables to the principal components.
(TIF)

Figure S3 **Robust principal component analysis (PCA) of log-transformed cytokine and chemokine secretion in PBMCs stimulated with ES antigen.** *Footnotes:* The principal component scores for individuals mono- (•) and co-infected (▴) with hookworm are shown. The respective mean values are shown as open symbols, with 95% confidence ellipses (*p* value for bivariate *T*2 test is 0.08). The arrows show the strongest loadings, i.e. contributions of the original variables to the principal components.
(TIF)

Table S1 **Correlations between hookworm-specific antibody responses and hookworm egg counts in mono- and co-infected individuals.** *Footnotes:* ^$^ Indicated are correlation coefficients and calculated *p*-values for statistical differences. **^#^** Statistically significant correlations in each group are highlighted in bold numbers.
(DOC)
