# Supplementary figures and images for: Necator americanus and Helminth Co-Infections: Further Down-Modulation of Hookworm-Specific Type 1 Immune Responses
Source: PLoS Negl Trop Dis. 2011 Sep 6;5(9):e1280. doi: 10.1371/journal.pntd.0001280 (PMC3167770; doi:10.1371/journal.pntd.0001280)

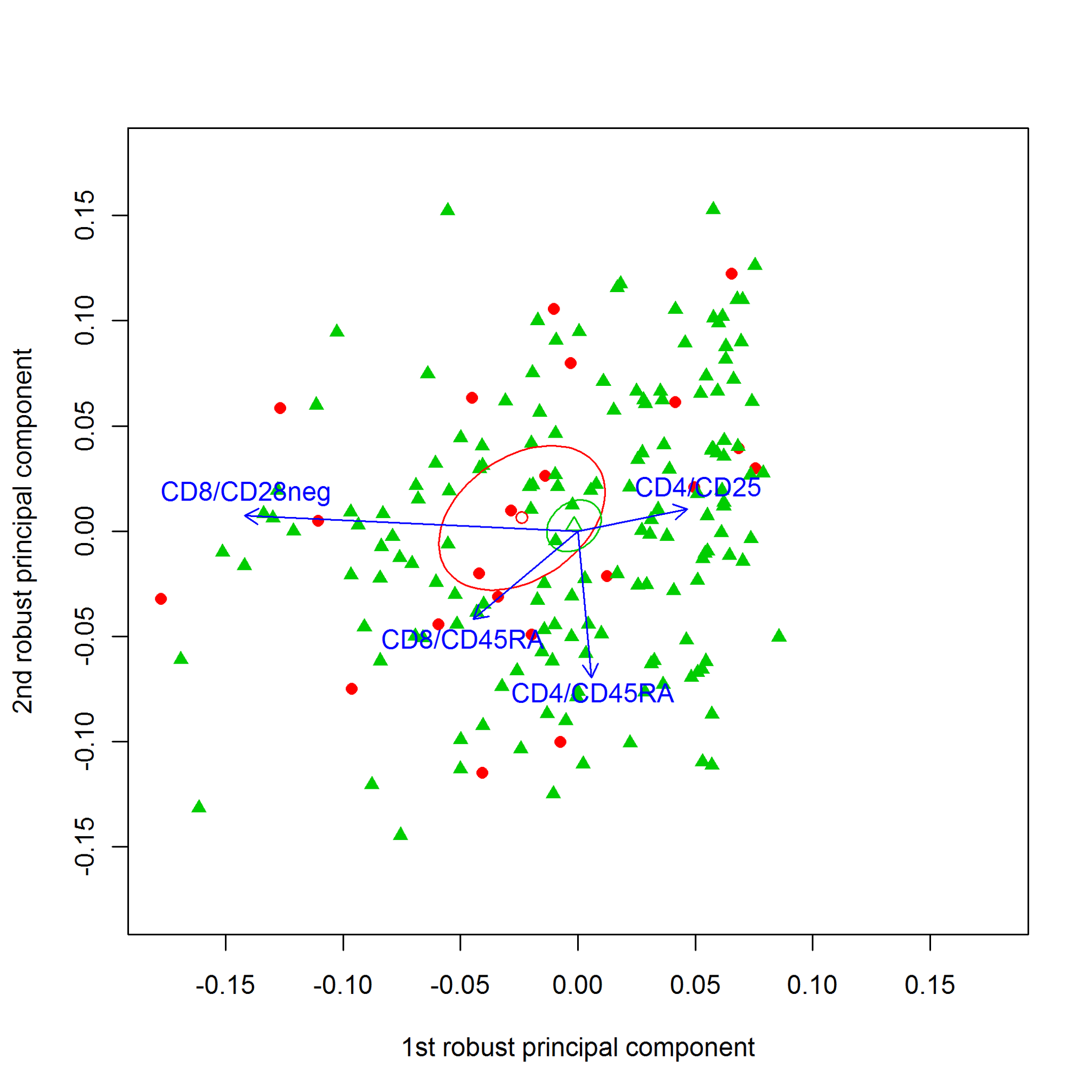

Supplement: Figure S1 — Robust principal component analysis (PCA) of ex vivo lymphocyte cell surface markers in PBMCs. Footnotes: The principal component scores for individuals mono- (•) and co-infected (▴) with hookworm are shown. The respective mean values are shown as open symbols, with 95% confidence ellipses (p value for bivariate T2 test is 0.23). The arrows show the strongest loadings, i.e. contributions of the original variables to the principal components. (TIF) [file pntd.0001280.s001.tif]

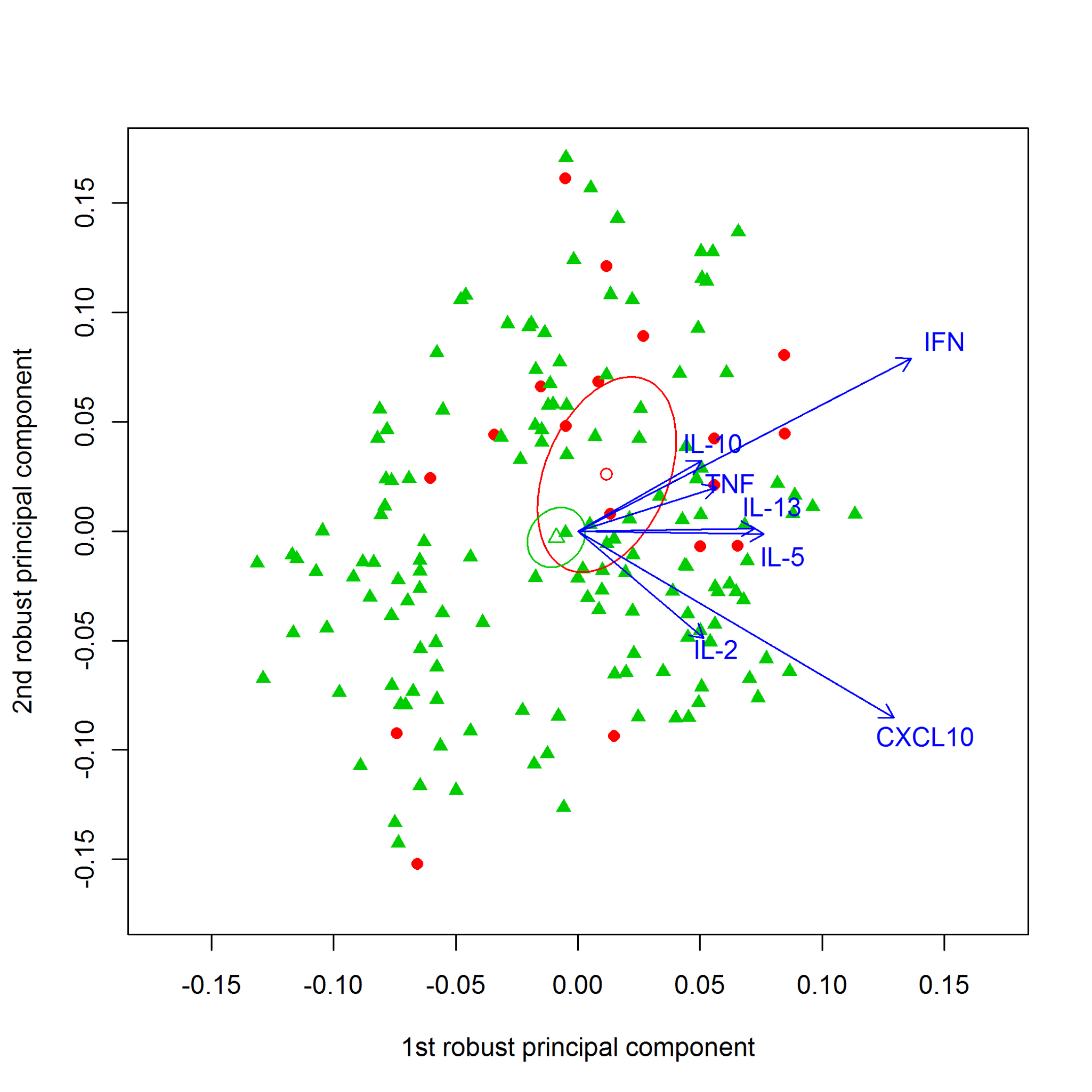

Supplement: Figure S2 — Robust principal component analysis (PCA) of log-transformed cytokine and chemokine secretion in PBMCs stimulated with AE antigen. Footnotes: The principal component scores for individuals mono- (•) and co-infected (▴) with hookworm are shown. The respective mean values are shown as open symbols, with 95% confidence ellipses (p value for bivariate T2 test is 0.13). The arrows show the strongest loadings, i.e. contributions of the original variables to the principal components. (TIF) [file pntd.0001280.s002.tif]

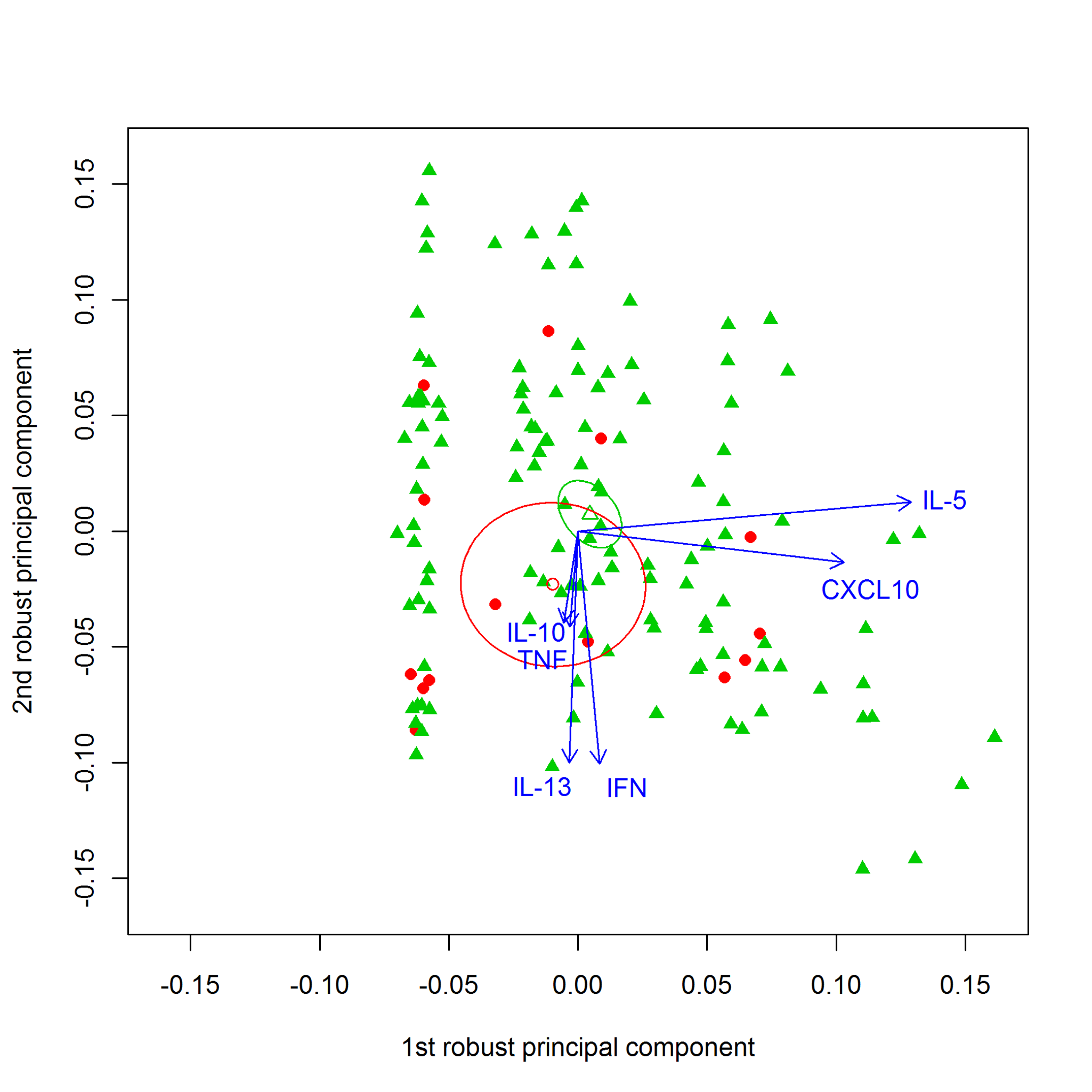

Supplement: Figure S3 — Robust principal component analysis (PCA) of log-transformed cytokine and chemokine secretion in PBMCs stimulated with ES antigen. Footnotes: The principal component scores for individuals mono- (•) and co-infected (▴) with hookworm are shown. The respective mean values are shown as open symbols, with 95% confidence ellipses (p value for bivariate T2 test is 0.08). The arrows show the strongest loadings, i.e. contributions of the original variables to the principal components. (TIF) [file pntd.0001280.s003.tif]
