# Supplementary material for: Necator americanus and Helminth Co-Infections: Further Down-Modulation of Hookworm-Specific Type 1 Immune Responses
Source: PLoS Negl Trop Dis. 2011 Sep 6;5(9):e1280. doi: 10.1371/journal.pntd.0001280 (PMC3167770; doi:10.1371/journal.pntd.0001280)
Supplement: Table S1 — Correlations between hookworm-specific antibody responses and hookworm egg counts in mono- and co-infected individuals. Footnotes: $ Indicated are correlation coefficients and calculated p-values for statistical differences. # Statistically significant correlations in each group are highlighted in bold numbers. (DOC) [file pntd.0001280.s004.doc]

**Table S1.** **Correlations between hookworm-specific antibody responses and hookworm egg counts in mono- and co-infected individuals.**

| Antigen | Antibody  classes and sub-classes  (n missing) | Mono-infected,  n=25 |  | Co-infected,  n=195 |  |
| --- | --- | --- | --- | --- | --- |
|  |  | Correlation coefficient,^$^ | *p-*value*^#^* | Correlation coefficient, ^$^ | *p-*value*^#^* |
| L3 | IgG1 (17) | -0.25 | 0.244 | 0.11 | 0.174 |
|  | IgG3 (17) | -0.12 | 0.598 | 0.05 | 0.562 |
|  | IgG4 (17) | 0.11 | 0.619 | **0.26** | **0.001** |
|  | IgE (7) | 0.00 | 0.986 | 0.11 | 0.175 |
| AE | IgG1 (16) | -0.21 | 0.337 | 0.12 | 0.120 |
|  | IgG3 (16) | -0.12 | 0.560 | 0.09 | 0.249 |
|  | IgG4 (16) | 0.23 | 0.288 | **0.40** | **<0.001** |
|  | IgE (7) | 0.16 | 0.478 | 0.10 | 0.180 |
| ES | IgG1 (17) | -0.05 | 0.810 | 0.01 | 0.945 |
|  | IgG3 (17) | -0.18 | 0.398 | 0.06 | 0.445 |
|  | IgG4 (17) | 0.22 | 0.317 | **0.21** | **0.007** |
|  | IgE (7) | -0.11 | 0.588 | 0.00 | 0.998 |

*Footnotes:*

^$^ Indicated are correlation coefficients and calculated *p*-values for statistical differences.

**^#^** Statistically significant correlations in each group are highlighted in bold numbers.
